# Auricular acupuncture for primary care treatment of low back pain and posterior pelvic pain in pregnancy: study protocol for a multicentre randomised placebo-controlled trial

**DOI:** 10.1186/1745-6215-15-288

**Published:** 2014-07-16

**Authors:** Jorge Vas, José Manuel Aranda-Regules, Manuela Modesto, Inmaculada Aguilar, Mercedes Barón-Crespo, María Ramos-Monserrat, Manuel Quevedo-Carrasco, Francisco Rivas-Ruiz

**Affiliations:** 1 Pain Treatment Unit. Doña Mercedes Primary Health Care Centre, Segovia s/n, Dos Hermanas 41701, Spain; 2 Carlos III Health Institute, Network of Research in Health Services in Chronic Diseases (REDISSEC), Sinesio Delgado 4, Madrid 28029, Spain; 3 San Andrés Torcal Primary Health Care Centre, José Palanca s/n, Málaga 29003, Spain; 4 El Lugar Primary Health Care Centre, Jesús Nazareno s/n, Chiclana 11130, Spain; 5 Mallorca Cancer Registry. Balearic Islands Public Health Department, Hospital Psiquiàtric, Camí de Jesús 40, Palma 07010, Spain; 6 Vélez Sur Primary Health Care Centre, Fernando Vivar s/n, Vélez-Málaga 29700, Spain; 7 Support Research Unit, Costa del Sol Hospital, Autovia A-7, Km 187, Marbella 29603, Spain

## Abstract

**Background:**

About 45% of all pregnant women suffer low back pain and/or pelvic girdle pain (LBPGP). This study seeks to evaluate the effect of auricular acupuncture on LBPGP compared with placebo auricular acupuncture and with standard obstetric care in the field of primary health care.

**Methods and design:**

This study will be a four-parallel-arm, multicentre, randomised, placebo-controlled trial. A total of 212 pregnant women (24 to 36 weeks’ gestation), aged at least 17 years, with LBPGP, will be randomly assigned to the verum auricular acupuncture plus standard obstetric care group (VAAc), to the non-specific auricular acupuncture plus standard obstetric care group (NSAAc), to the non-specific placebo auricular acupuncture plus standard obstetric care group (PAAc), or the standard obstetric care group (SOC). The VAAc, NSAAc, and PAAc groups will receive treatment at three auricular acupuncture points (specific points for the VAAc group or non-specific ones for the NSAAc and PAAc groups), once a week for 2 weeks; the SOC group will receive only standard obstetric care during the same period. The primary outcome will be the reduction in pain intensity, according to the visual analogue scale (iVAS), at 2 weeks after the start of treatment. The secondary outcomes will be functional status with respect to LBPGP (according to the Roland-Morris disability questionnaire), health-related quality of life (SF12) at 2 weeks after the start of treatment, and iVAS at 12 and 48 weeks postpartum.

**Discussion:**

This trial will implement a high-quality methodology and may provide evidence for the efficacy, safety, and specificity of auricular acupuncture as a treatment for pregnant women with LBPGP.

**Trial registration:**

Current Controlled Trials ISRCTN41033073 (date 20/03/2014).

## Background

Musculoskeletal pain in pregnant women is often regarded as something that is transient, physiological, and self-limiting, but low back pain (LBP) and pelvic girdle pain (PGP) during pregnancy are associated with an increased risk of suffering these pains in future pregnancies and of the pain becoming chronic [1].

Low back pain is usually defined as occurring between the twelfth rib and the gluteal fold. Pelvic girdle pain is experienced between the posterior iliac crest and the gluteal fold, particularly in the vicinity of the sacroiliac joints. The pain may radiate to the back of the thigh and may occur in isolation or be associated with pain in the symphysis pubis [2]. Opinions are divided as to whether LBP and PGP should be associated or differentiated. According to some experts, they can be differentiated clinically, responding to different treatments and having different risk factors [3]. However, studies have so far failed to distinguish reliably between the two, and LBP is very likely to be a subgroup of the pelvic pain associated with pregnancy [4]. In fact, similar pathophysiological mechanisms – joint laxity, increased lumbar lordosis, and muscle weakness – have been suggested to be at the cause of both conditions [5-7].

About 45% of all pregnant women suffer from LBP and/or PGP (LBPGP) [8]. The prevalence appears to be somewhat higher in the Nordic countries [9], and higher still in Spain, where the prevalence at 4 weeks is estimated to be 71.3% for LBP and 64.7% for PGP [10]. LBPGP usually begins from week 18 of pregnancy, and although it may appear in the first quarter, the peak intensity is from weeks 24 to 36 [8].

There are three major risk factors for the development of LBPGP during pregnancy: strenuous work, the presence of LBP before pregnancy, and the development of LBPGP during previous pregnancies [8]. On the other hand, the use of contraceptives, the period of time elapsed since the last pregnancy, maternal height and weight, smoking, and age [2] are not usually considered risk factors, and neither the use of epidural or spinal anaesthesia or analgesia seem to be relevant [11].

LBPGP hampers women’s everyday activities, such as getting up, turning over in bed, sitting down, walking, dressing and undressing, lifting, and carrying small objects. It can also impede sexual relations, render subjects unable to work [12,13], and, in general, negatively affect their quality of life [14].

LBPGP during pregnancy is usually diagnosed in primary care, taking into account the patient’s medical history and the results of a physical examination, which will seek to exclude other causes of pain and to assess the degree of disability, as well as identifying any warning signs that could indicate the presence of inflammatory, infectious, traumatic, neoplastic or degenerative processes, that would require referral for study by other diagnostics [15]. It is unclear whether there exists any effective preventive intervention against LBPGP in pregnancy; prior studies on this question are conflicting [16,17] and, in any case, it appears that only pregnant women with previous LBP might benefit from such treatment [17].

Various therapeutic options have been proposed for pregnant women with LBPGP. The use of paracetamol is considered safe but not very effective [18]. Non-steroidal anti-inflammatory drugs do not appear to be associated with foetal malformation before 12 weeks of pregnancy [19] – although before this gestational age, few women need such medication – but are contraindicated in the third trimester of pregnancy, being associated with an increased risk of premature closure of the foetal ductus arteriosus and with oligohydramnios [20]. The association between opioid use and teratogenicity is a complicated question because the available data are contradictory and incomplete. Although data from previous studies suggest that there is no significant additional risk of congenital anomalies, there is undoubtedly a slight increase in certain heart defects, spina bifida, and, possibly, gastroschisis [21].

Prominent among non-pharmacological interventions are education, physical therapy, exercises, transcutaneous nerve stimulation, and acupuncture. The provision of specific education for pregnant women and of advice on pain-prevention strategies appears to reduce the sick leave needed by pregnant women with LBP, but not by those with PGP [3]. A recent systematic review [22] concluded that pregnant women with LBP who performed specifically adapted strengthening exercises in programmes of pelvic tilt exercises and water gymnastics reduced the intensity of LBP and the need for pain-related sick leave to a greater degree than was achieved by women who received only standard antenatal care. The same authors reported that specially designed pillows reduce pain more than normal ones, but these are not available in Spain.

Studies and reviews have supported the use of acupuncture by pregnant women with LBPGP [22,23]. Thus, it has been reported that acupuncture and stabilising exercises more effectively relieve PGP compared to standard antenatal care, and that acupuncture can provide more relief for night-time pain than is obtained by exercise alone. In a study of pregnant women with pelvic and back pain, acupuncture was found to be more effective than physiotherapy in reducing pain intensity [24]. Another study reported that 60% of pregnant women who received acupuncture suffered less intense pain, compared with 14% of those who received standard [25] antenatal care. A recent study found that acupuncture performed on women with LBPGP is more effective when applied from week 26 than during week 20 of pregnancy [26]. The adverse effects observed are minor and temporary, such as bruising, pain or swelling in the puncture site, asthenia, or nausea [25]. A recent survey showed that over 60% of pregnant women with LBPGP would accept some form of complementary therapy, including acupuncture [27].

The analgesia produced by acupuncture is caused by complex neurohormonal mechanisms involving endogenous opioids and monoamines [28], with evidence of a sustained depression of the neurons in the spinal dorsal horn [29]. Some of the effects of acupuncture may be partially explained within a conventional neurophysiological model, but there remain certain empirically-supported aspects that resist conventional explanation. Furthermore, the use of auricular acupuncture has been shown to be effective in reducing LBP in non-pregnant women [30]. Just one study published to date has evaluated the results of auricular acupuncture in pregnant women, and this paper reported that the insertion of needles in the ear for a period of one week reduces the pain and disability suffered by pregnant women with LBPGP [31]. This study was conducted in the USA and in a hospital setting. However, there have been no previous well-designed studies to evaluate the effects of auricular acupuncture in pregnant women with LBPGP compared with placebo auricular acupuncture, on the one hand, and with standard obstetric care on the other, in the field of primary health care and carried out by midwives trained in this technique. In fact, these healthcare providers have made significant contributions to studies in this field conducted by our research team [32].

### Hypothesis and study goals

Our clinical hypothesis is that acupuncture applied via pressure needles inserted in the auricle (VAAc), associated with standard obstetric care (SOC), can reduce the pain experienced by pregnant women in the lower back and/or the posterior pelvic girdle, to a greater extent than is achieved by SOC alone in the field of primary health care. Additionally, the application of this technique improves patients’ functional status and health-related quality of life, and moderates the consumption of drugs used in conventional therapy, thus reducing the associated iatrogenic effects without provoking significant iatrogenesis in itself. Secondly, VAAc applied together with SOC has specific effects, achieving a greater reduction in the LBPGP suffered by pregnant women than that achieved with the application of pressure needles at non-specific pressure points (NSAAc) or with placebo needles at non-specific points (PAAc).

The main aim of this study is to evaluate the efficacy achieved in terms of reduction in pain intensity (0 to 100 mm visual analogue scale) at 2 weeks after starting treatment. Our secondary aims are: i) to evaluate the efficacy in terms of improved functional status with respect to LBPGP (according to the Roland-Morris disability questionnaire) at two weeks after starting treatment; ii) to evaluate the efficacy in terms of improved health-related quality of life (SF12) at two weeks after starting treatment; iii) to evaluate the efficacy in terms of improvement perceived by the patient with LBPGP at two weeks after starting treatment; iv) to evaluate the impact on temporary occupational incapacity of pregnant women with LBPGP; v) to evaluate the efficacy in terms of reduced consumption of analgesic medication at 2 weeks after starting treatment; vi) to evaluate the presence of LBPGP at 12 weeks postpartum; vii) to evaluate the presence of LBPGP at 48 weeks postpartum; and viii) to estimate the placebo effect and the specific and non-specific effects of VAAc, NSAAc, and PAAc.

## Methods/design

### Design

**Figure 1:**
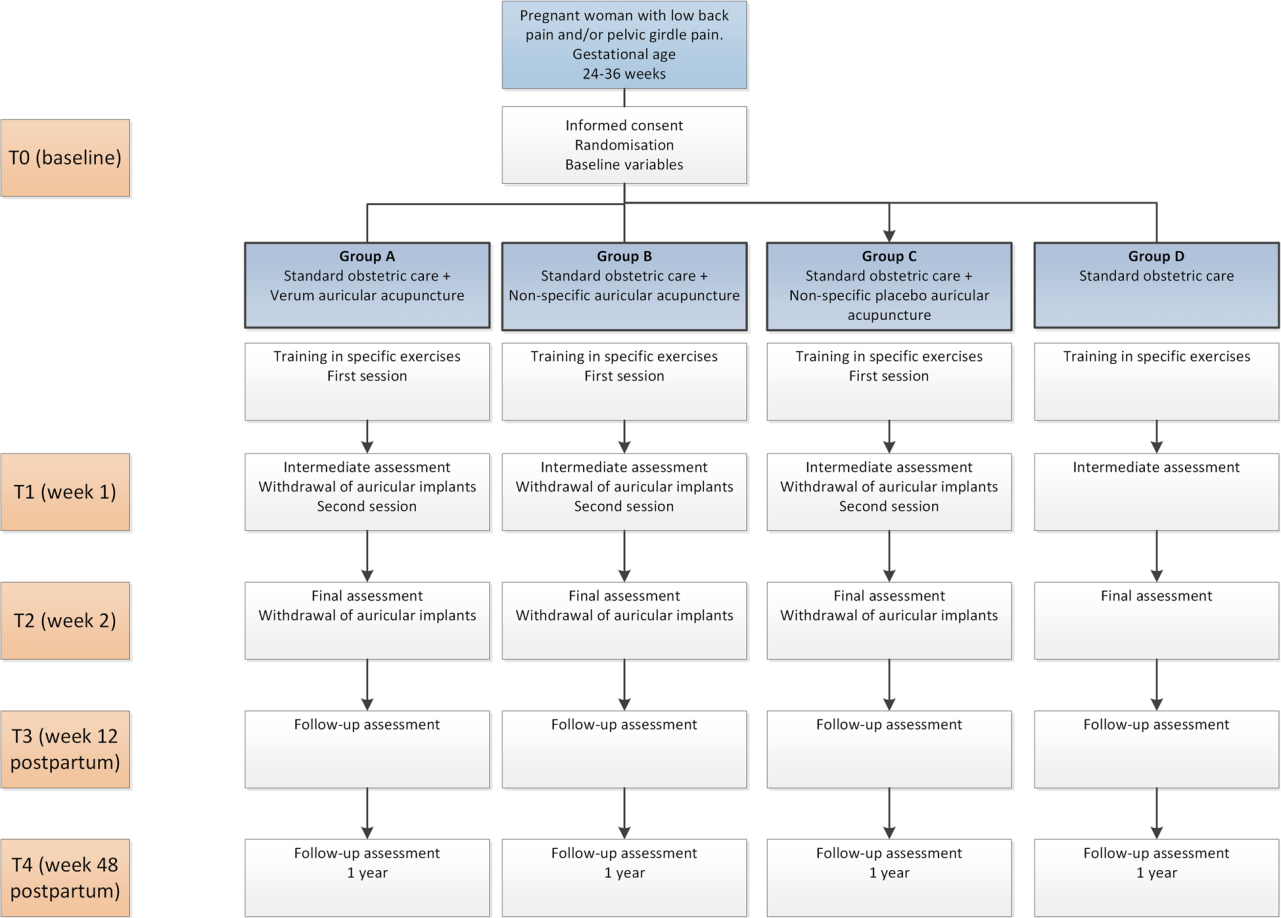
Trial flow.

Multicentre prospective randomised controlled study, with four parallel arms, to compare acupuncture with pressure needles inserted in the ear (VAAc) together with SOC; non-specific auricular acupuncture plus SOC (NSAAc); non-specific placebo acupuncture plus SOC (PAAc); and SOC alone (Figure 1). The results will be analysed by statisticians blinded with regard to the allocation of patients to the different treatment groups.

### Study period

February 2014 to December 2016.

### Study scope and subjects

The study population will be composed of pregnant women (24 to 36 weeks’ gestation), aged 17 years or more, referred by their family physicians from the 21 primary care centres participating in the study, belonging to the Andalusian Public Health System and located in the provinces of Seville and Málaga, diagnosed with pregnancy-related LBPGP and who have not previously received auricular acupuncture. Women with LBPGP that began before the pregnancy, or presenting any of the ‘red flags’, which would require examination to rule out the possible presence of LBPGP secondary to inflammatory, infectious, traumatic, neoplastic, or degenerative processes, together with women receiving anticoagulant treatment or presenting dermatitis of the auricle, will be excluded from the study.

The women will be given the following information: “*This study is being conducted to compare the effects of standard obstetric care either alone or in conjunction with one of three types of stimulation of the ear (auricular acupuncture). One of these is similar to traditional Chinese auricular acupuncture, while the others do not follow these principles. One is a placebo intervention, but all have been associated with positive outcomes in various clinical studies*”. The women will also be informed of the potential risks associated with different types of auricular acupuncture (infection, pain at the puncture site, fainting, bruising) and that they may terminate their participation in the study at any time without any penalty or loss of the benefits to which they are entitled. All study participants must sign the informed consent form.

### Randomisation and blinding procedures

The four study arms will be randomised centrally, at the Costa del Sol Hospital, Research Support Unit, to achieve a 1:1:1:1 allocation ratio, stratified by healthcare centre. The health professionals taking part in the study will not be involved in the randomisation process. Patients who meet the inclusion criteria and give their signed informed consent will be included in the study. After inclusion, the researcher will contact the randomisation centre, where the patient’s data will be recorded, and the midwife will be informed, by telephone and by fax, of the patient’s allocation to one of the four study arms. This procedure ensures that randomisation will not be influenced by any of the researchers involved. The patients assigned to the VAAc, NSAAc, and PAAc groups will be blinded with respect to the treatment allocated to them.

### Treatment

A 4-hour workshop will be organised for all the midwives participating in the study to demonstrate the implementation of the technique, the obstetric care to be provided, and the study records to be kept. The workshop will be given by two physicians who are specialists in acupuncture and have over 10 years’ clinical experience. At one month after initiating the study, a test will be applied to confirm that the skills acquired in the workshop are retained.

#### Standard obstetric care (SOC)

The SOC for the treatment of LBPGP in pregnancy will include an explanation of its cause and of recommended self-care procedures, both to prevent pain and to reduce its intensity, together with training in specific stretching exercises for the back and the hamstrings. In addition, the women participating in the study will be recommended to use paracetamol and/or visit their family doctor if the pain intensity becomes severe.

#### Verum auricular acupuncture (VAAc)

**Figure 2:**
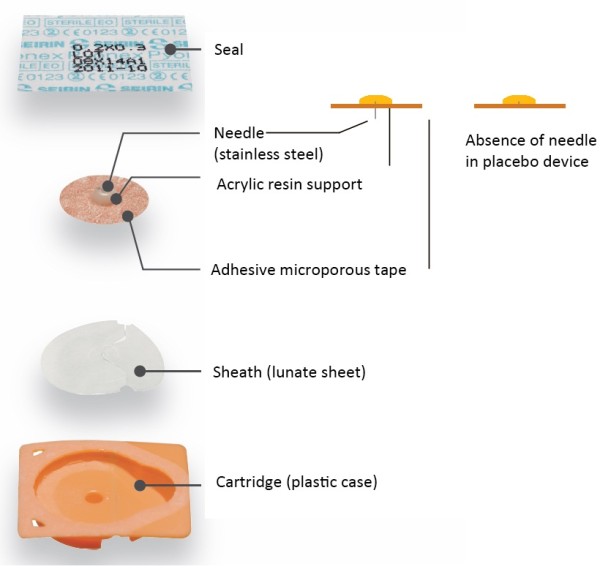
Auricular device used for the verum auricular acupuncture and non-specific auricular acupuncture (New Pyonex) groups and for the placebo auricular acupuncture group.

**Figure 3:**
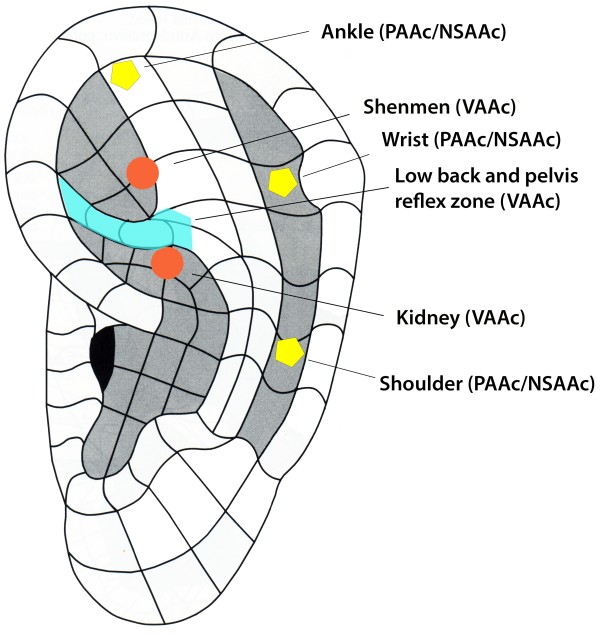
Specific and non-specific auricular acupuncture points used for the different study groups.

Auricular pressure needles 1.5 mm long and 0.20 mm in diameter (Pyonex Seirin, Shizuoka, Japan) (Figure 2) will be applied to two standardised points (Shenmen and Kidney), and at a reflex point in the region of the auricle that classically represents the lumbar or sacral regions [33], and which will be detected by means of a probe calibrated at 250 g of pressure (Figure 3). The points will be located in a single ear, preferably the one on the side of the body corresponding to the location of the pain; if the pain is bilateral, the most sensitive ear will be determined by using a probe to exert a pressure of 250 g. Before placing the implants, the ear will be disinfected using chlorhexidine. The patients will be instructed not to exert pressure on the implants at any time.

#### Non-specific auricular acupuncture (NSAAc)

The needles used in the non-specific auricular acupuncture group will be the same as in the VAAc group, but they will be applied at auricular points that are non-specific for LBPGP, and which instead correspond to anatomic locations in the ankle, wrist, and shoulder (Figure 3).

#### Placebo acupuncture (PAAc)

The devices used in the placebo group will be supplied by Seirin (Pyonex Placebo, Seirin, Shizuoka, Japan) and by 3B Scientific (Spain). They will be identical to those used in the VAAc group, but without the needle [34] (Figure 2), and will be applied at the same non-specific points as for the NSAAc group.

#### Data sourcing and compilation

We have designed a data form on which the variables of interest should be entered by the researcher at each centre. The baseline assessment (T0), intermediate result at one week (T1), end result at two weeks (T2), follow-up at 12 weeks postpartum (T3), and follow-up at 48 weeks postpartum (T4) will be performed by the midwives taking part in the study, who may also instruct the patients on how to complete the self-administered questionnaire. The information will be electronically recorded and stored, at a single coordinating point for each province, in a database for subsequent statistical analysis. Data collection will be conducted in person, at times T0, T1, T2, and T3. Only the evaluation at T4 will be carried out by telephone. Patient confidentiality will be maintained by removing patient identity data from the database. Figure 1 summarises the flow plan for the study.

### Variables

The primary outcome will be the reduction in pain intensity, according to the visual analogue scale (iVAS), at 2 weeks after the start of treatment. The secondary outcomes will be functional status with respect to LBPGP (according to the Roland-Morris disability questionnaire) and health-related quality of life (SF12) at 2 weeks after the start of treatment, iVAS score at 12 and 48 weeks postpartum, presence of temporary occupational incapacity, reduction in the consumption of analgesic medication at 2 weeks, and presence of pain at 12 and 48 weeks postpartum.

The obstetric data, based on the patient’s health record, will consist of gestational age (weeks), number of previous pregnancies, number of children, previous caesarean sections, and whether epidural anaesthesia was required. The personal data compiled by the midwives participating in the study will be patient’s age (years), weight before pregnancy and present weight (kg), height (cm), body mass index before pregnancy (calculated as weight/height^2^), educational level (none, primary education, secondary education, high school, university), and employment status (employed, non-employed, type of work and, where appropriate, the reasons for not working). In addition, any previous pregnancy-related LBPGP will be recorded. If the patient is temporarily unable to work, the days elapsed since this condition began will be noted, and its follow-up at T2, T3, and T4. The data form will also record the consumption of healthcare resources since the beginning of the LBPGP (number of times treated by the family doctor, number of times emergency treatment needed at the health centre or hospital, number of visits to a medical specialist, private doctor, company doctor, or non-conventional healthcare professional).

In addition, data will be recorded for pain intensity, location (lower back, posterior pelvis or both, and whether pain is radiating to the leg), onset, and frequency of pain (occasional days, nearly every day, every day). Pain intensity will be measured by a visual analogue scale (iVAS) at T0, T1, and T2, as well as at the follow-up assessments T3 and T4. There is ample evidence supporting the validity of the iVAS for pain intensity, and many studies have corroborated its construct validity [35] and reliability [36,37]. It is a simple and fast method to assess subjective pain intensity. The patient is asked to mark on a 100-mm scale from 0 (no pain) to 100 (worst pain imaginable) the intensity of back pain by day (iVASd) and night (iVASn). The primary outcome, the change in pain intensity (iVAS) at 2 weeks after the start of treatment (T2), will be determined from the maximum value of the daytime pain measurements (iVASd) or the night-time ones (iVASn) at 2 weeks after starting treatment. There is evidence that reductions of greater than 35 mm on this scale are associated with an improvement perceived by the patient [38].

The women taking part in the study will be asked to complete the Roland-Morris disability questionnaire to assess the level of disability provoked by LBPGP at T0 and at T2. This self-administered questionnaire has been validated for use in Spain [39] and consists of 24 questions or statements related to incapacity caused by LBP. Respondents are asked to award one point for each statement that corresponds to their current situation. Scoring is quick and easy: the total score awarded may range from 0 points (no incapacity caused by LBP) to 24 (maximum incapacity). Changes in health-related quality of life are valued according to the SF-12 v.2 self-administered questionnaire. This is a generic questionnaire, derived from SF-36, which has been validated for use in Spain [35]. Version 2 allows the researcher to calculate the respondent’s quality of life in eight domains (physical function, physical role, pain, general health, vitality, social function, emotional role, mental health) and two summary components (physical and mental). A version with six items, to enable the calculation of utilities, is currently being validated. In our study, this would enable a cost-effectiveness analysis to be conducted.

The presence of anxiety and/or depression will be screened and assessed at T0 and T2 by the Spanish version of the Goldberg Depression Inventory [40]. This consists of two scales, one for anxiety and one for depression, each with nine dichotomous items (yes/no answers). A separate score is given for each scale, with one point for each affirmative answer. In this Inventory, which may be applied by the researcher or be self-administered, the patient is asked whether in the last 2 weeks any of the symptoms referred to in the questions have occurred. Symptoms lasting less than 2 weeks or of only mild impact are not counted. The questionnaire can be applied even by non-medical staff, and no standardisation is required. Each of the subscales is divided into four initial screening items to determine whether a mental disorder is likely to be present, and a second group of five items will be asked only if positive responses are given to the screening questions (scores of ≥2 on the anxiety subscale and ≥1 on the depression subscale). Cut-off points are ≥4 for the anxiety scale and ≥2 for the depression scale. There is a clear improvement in the sensitivity of this Inventory with increasing severity of the psychopathology, with higher scores being obtained, which can provide a separate dimensional measure of the severity of each disorder. Raising the cut-off points to ≥5 and ≥3 improves the specificity and the discriminatory power of the scales, but slightly reduces their sensitivity (specificity 93%, sensitivity 74%).

At the end of treatment (T2), the patient’s perceived improvement will also be assessed. A 7-point Likert scale has been recommended to assess the perceived improvement of a patient with non-specific LBP [41], but opinions differ regarding the categories to be used; we opted for the suggestion made by Hudak and Wright [42]: How satisfied are you with the results of your recent treatment for low back pain and/or posterior pelvic pain? 1 = very satisfied, 2 = very satisfied, 3 = somewhat satisfied, 4 = indifferent (about equally satisfied and dissatisfied), 5 = somewhat dissatisfied, 6 = very dissatisfied, and 7 = extremely dissatisfied.

We will also evaluate the consumption of analgesic medication, whether or not prescribed by the patient’s doctor, both at the time of randomisation (T0) and at final assessment (T2), according to a 4-point Likert scale of 0–3: (0) = none, (1) = less than the usual dose, (2) = usual daily dose, and (3) = greater than the usual dose. We will also record the names of the medications taken and the daily dose in each case.

The Treatment Credibility Scale [43] will be used to evaluate patients’ expectations and the credibility of the treatments provided in the study. The original Borkovec and Nau scale [44] with four items will be applied, assessing the following questions on a continuous VAS of 0 to 10 (0 to 10, from totally disagree to totally agree): (1) = Are you confident that this treatment can alleviate your pain? (2) = Does it seem a logical treatment? (3) = Would you recommend this treatment to a friend or family member who was suffering the same problem? (4) = Do you think this treatment would be an option for treating other problems? Questions 1 and 2 will be assessed after the first treatment session, and questions 3 and 4, after 2 weeks.

After the treatment has concluded, the blinding of the patients’ allocation to one of the two auricular acupuncture groups (real or placebo) will be verified by the following dichotomous question: Do you think you were assigned to the real auricular acupuncture group or to the placebo group?

During the study, side effects and possible adverse reactions resulting from the treatment will be recorded.

### Sample size

Taking Wang et al. [31] as a reference, for the primary purpose of the study, namely to compare the change in pain intensity, assessed on an iVAS, between the baseline assessment and the value at 2 weeks after the intervention, a difference of over 20 mm between the VAAc group and the SOC group will be considered statistically significant. For a known standard deviation of 25 mm for each group, a type I (alpha) error of 0.01 and a type II (beta) error of 0.10, 47 patients per group will be required. Including the patients for the PAAc and NSAAc arms, and with an estimated 10% loss to intervention follow-up, a total of 212 patients (53 per group) will need to be assessed.

### Statistical analysis

A descriptive analysis will be performed to assess the comparability of the four groups with regard to baseline demographic information, prognostic variables, and the evaluation of credibility, using measures of central tendency and dispersion for the quantitative variables and frequency distributions for the qualitative ones.

To determine the primary study outcome (change in pain intensity at 2 weeks) and secondary outcomes (change at 2 weeks in functional status with respect to pain, quality of life, and improvement perceived by the patient), a crude analysis will be performed to compare the mean differences between baseline values and at 2 weeks, for the VAAc group and the SOC group, using the Student *t*-test for independent samples or the Mann-Whitney U test if the samples do not present a normal distribution (determined by the Shapiro-Wilk test). Subsequently, simple and multivariate linear regression analyses will be performed, using the same outcome variables, to adjust for unbalanced independent variables, including the treatment group in the first pass, and determining regression coefficients with the corresponding 95% confidence intervals and the coefficient of determination. The main analysis will be by intention-to-treat, but a per protocol analysis will also be performed.

To determine the secondary outcomes of presence of temporary occupational incapacity, reduction in the consumption of analgesic medication at 2 weeks, and presence of pain at 12 and 48 weeks postpartum, the VAAc and SOC groups will be compared, using the χ^2^ test with continuity correction or Fisher’s exact test when the expected values are below 5. Then, simple and multivariate logistic regression models will be obtained, with the same outcome variables, to adjust for unbalanced independent variables, including the treatment group in the first pass, and the relative risk will be determined, with 95% confidence intervals.

Both the quantitative outcome variables (mean differences) and the qualitative ones (difference in proportions) will be expressed with the respective 95% confidence intervals and intention-to-treat analysis criteria will be used (last known value for losses-to-study for the quantitative outcome variables, and penalisation of results in the experimental treatment for the qualitative variables). To assess the specificity of the effect of auricular acupuncture, previous analyses comparing the VAAc treatment group versus PAAc and NSAAc will be repeated. In each test, the bilateral statistical significance limit of *P* <0.01 will established. An intermediate analysis will be performed when complete tracking is concluded for half of the sample population, setting the level of bilateral statistical significance at *P* <0.005, this being a necessary penalisation to minimise type I errors.

### Ethical issues

The ethical validity of this study has been examined by the Regional Clinical Trials Committee. It was subsequently approved by the Research Ethics Committee (Comité de Ética de la Investigación Área Sanitaria Sevilla Sur, 20130429). The project has received funding from a public national call for proposals, awarded on a competitive basis. The study complies with the Declaration of Helsinki and subsequent updates (to the 2008 revision) and takes into account the principles set out in the Council of Europe Convention on Human Rights and Biomedicine, as well as the requirements established by Spanish legislation regarding biomedical research, the protection of personal data, and bioethics. During the course of the study, audits will be performed as required by the above-mentioned Research Ethics Committee and by the hospital’s Quality Commission, independently of any external audits (source of research funding) that may be deemed necessary. The forms, case report data, and computer data files will be identified with codes, not the names of the patients, in order to protect personal data. Statistical analysis will be performed by third parties who will be unaware of the source data (blinded analysis).

## Discussion

When auricular acupuncture is practiced according to the principles of Traditional Chinese Medicine, it is an individualised treatment. In this study we have designed a semi-standardised treatment protocol that approximates standard clinical practice, which is sufficiently flexible and straightforward so that it can be performed by midwives without prior training, and which enables reproducibility.

As this experimental study is designed to be applied by medical personnel whose training is limited to an intensive 4-hour course in the technique, obviously the auricular acupuncture treatment cannot be performed to its full extent. For this reason, we have limited the number of points where acupuncture needles will be applied, and therefore the therapeutic effect obtained may be less than that otherwise to be expected. This limitation will produce an effect contrary to the study hypothesis.

Moreover, the patients assigned to the PAAc and NSAAc groups, due to the healthcare provided and the effects of dermatological stimulation, however minimal, may achieve some positive results. This effect, too, is contrary to the study hypothesis, but to a certain extent will reflect the non-specific effect of the intervention.

A double-blind study cannot be carried out because the therapist must know which treatment is being applied. We have attempted to minimise the consequences of this situation by ensuring that the professional who provides the treatment does not intervene in the assessment of the outcome. Furthermore, the blinding of the evaluators and the patients is designed to be highly effective. In addition, a question will be included to determine the patients’ perceptions regarding the type of auricular acupuncture received. Since the main outcome variable and many of the secondary ones will be recorded by means of a self-administered questionnaire no added bias will be caused by the intervention of the midwife in this area.

The patients will be asked not to receive any alternative treatment. Nevertheless, although the women in the study population will be asked to confirm this, we cannot be absolutely certain of compliance. Further, an important limitation may arise from the patients’ lack of adherence to the prescribed treatment, as for various reasons they may fail to attend one or more treatment sessions. The main analysis will be by intention-to-treat, but a per protocol analysis will also be performed. The sample size was calculated on the assumption of a 10% drop-out rate, but it will be necessary to ensure there are no differential losses between the three treatment arms.

## Trial status

Currently in the patient-recruitment phase.

## Abbreviations

iVAS: Pain intensity, according to the visual analogue scale; iVASd: Pain intensity by day, according to the visual analogue scale; iVASn: Pain intensity by night, according to the visual analogue scale; LBP: Low back pain; LBPGP: Low back pain and/or pelvic girdle pain; NSAAc: Non-specific auricular acupuncture; PAAc: Non-specific placebo auricular acupuncture; PGP: Pelvic girdle pain; SF12: 12-Item short form health related quality of life survey; SOC: Standard obstetric care; VAAc: Verum auricular acupuncture.

## Competing interests

The authors declare that they have no competing interests.

## Authors’ contributions

JV conceived the study, designed the study protocol, sought funding and ethical approval, and wrote the manuscript. JMAR, MM, IA, MBC, and MQC made a substantial contribution to designing the individualised acupuncture treatment protocol, MRM made a critical review of the protocol. FR is responsible for the statistical analyses. All authors have critically reviewed and approved the final version of the manuscript. The corresponding author has final responsibility for the decision to submit for publication.
